# Serglycin-Deficiency Causes Reduced Weight Gain and Changed Intestinal Cytokine Responses in Mice Infected With *Giardia intestinalis*

**DOI:** 10.3389/fimmu.2021.677722

**Published:** 2021-07-08

**Authors:** Zhiqiang Li, Dimitra Peirasmaki, Staffan Svärd, Magnus Åbrink

**Affiliations:** ^1^ The Key and Characteristic Laboratory of Modern Pathogen Biology, College of Basic Medicine, Guizhou Medical University, Guiyang, China; ^2^ Department of Biomedical Sciences and Veterinary Public Health, Swedish University of Agricultural Sciences, Uppsala, Sweden; ^3^ SciLifeLab, Department of Cell and Molecular Biology, Uppsala University, Uppsala, Sweden

**Keywords:** serglycin proteoglycan, knockout mouse, infection, *Giardia intestinalis*, innate intestinal immunity

## Abstract

The proteoglycan serglycin (SG) is expressed by different innate and adaptive immune cells, *e.g.* mast cells, macrophages, neutrophils, and cytotoxic T lymphocytes, where SG contributes to correct granule storage and extracellular activity of inflammatory mediators. Here the serglycin-deficient (SG^−/−^) mouse strain was used to investigate the impact of SG on intestinal immune responses during infection with the non-invasive protozoan parasite *Giardia intestinalis*. Young (≈11 weeks old) oral gavage-infected congenic SG^−/−^ mice showed reduced weight gain as compared with the infected SG^+/+^ littermate mice and the PBS-challenged SG^−/−^ and SG^+/+^ littermate mice. The infection caused no major morphological changes in the small intestine. However, a SG-independent increased goblet cell and granulocyte cell count was observed, which did not correlate with an increased myeloperoxidase or neutrophil elastase activity. Furthermore, infected mice showed increased serum IL-6 levels, with significantly reduced serum IL-6 levels in infected SG-deficient mice and decreased intestinal expression levels of IL-6 in the infected SG-deficient mice. In infected mice the qPCR analysis of alarmins, chemokines, cytokines, and nitric oxide synthases (NOS), showed that the SG-deficiency caused reduced intestinal expression levels of TNF-α and CXCL2, and increased IFN-γ, CXCL1, and NOS1 levels as compared with SG-competent mice. This study shows that SG plays a regulatory role in intestinal immune responses, reflected by changes in chemokine and cytokine expression levels and a delayed weight gain in young SG^−/−^ mice infected with *G. intestinalis*.

## Introduction

*Giardia intestinalis* (also named *G. lamblia* or *G. duodenalis*) is a non-invasive protozoan intestinal parasite found worldwide. Infection with *G. intestinalis* mainly cause a self-limiting diarrheal-disease, *i.e.* giardiasis, in humans and other mammals (1). Although *Giardia-*infections often are asymptomatic, symptoms in affected patients include acute or chronic diarrhea, weight loss, and malabsorption (2). The parasite can be transmitted among domestic animals, pets, wild life, and humans. *Giardia* spp. secrete no known toxins, still infections contribute to more than 200 million human diarrhea cases per year (3) Since 1954, at least 132 water-borne outbreaks of giardiasis have been reported worldwide (4). Recent studies showed that *G. intestinalis* is a significant factor in the induction of food-borne disease, with reduced weight gain and growth stunting of young children in low-resource settings (5–7). Malnutrition due to *Giardia*-infections has also been replicated in mouse models (8, 9). When *G. intestinalis* attach to the microvillus brush border of the intestinal epithelial cells, the cells respond by producing chemokines and cytokines, which attracts immune cells to the intestinal submucosa (10–12). Both innate and adaptive immunity, with a mixed Th1/Th2/Th17 response profile, play significant roles in the host defence towards *G. intestinalis* (13–15). However, during infection *G. intestinalis* secretes a large number of immunomodulatory proteins, which possibly regulates the host intestinal immune responses (16–19).

The proteoglycan serglycin (SG) is mainly expressed in hematopoietic cells where SG promotes granule integrity, *e.g.* in mast cells, neutrophils, and cytotoxic T lymphocytes (20). Endothelial and epithelial cells also express SG, in which SG mainly contributes to the apical secretion of cytokines (21–23). The core protein SG carries long unbranched negatively charged disaccharide chains, *i.e.* anionic glycosaminoglycans (GAGs), and depending on cell type the GAGs attached to SG can be heparin, heparan sulfate, or chondroitin sulfate, for example. The GAGs of SG contribute a binding surface for cationic inflammatory mediators and the genetic ablation of SG showed mainly a granule storage deficiency of several cationic proteins: *e.g.* the mast cell specific proteases, the chymase mouse mast cell protease (mMCP)-4, the chymase/elastase mMCP-5, the tryptase mMCP-6, and the carboxypeptidase A3 (CPA3); the neutrophil elastase (NE); as well as granzyme B in cytotoxic T lymphocytes (20, 24–27). SG and the cationic mediators are believed to form functional complexes of physiological relevance (20), and thus, when released from cells can have impact locally but also systemically.

Previously we showed that the SG-deficient mice had increased intestinal neutrophil recruitment and NE activity, compared to wild type littermate mice, in response to the invasive nematode *T. spiralis* (28). With this notion in mind, and since different types of parasitic infections as well as the SG-deficiency may affect multiple innate and adaptive immune cells and mediators, we here studied the impact of SG on the small intestinal immune response during an experimental infection with the non-invasive protozoan parasite *G. intestinalis*. In young (≈11 weeks old) SG^+/+^, SG^+/−^ and SG^−/−^ congenic littermate mice we assessed weight and intestinal morphological changes, and analyzed serum levels of IL-6 as well as the transcriptional expression levels of intestinal cytokines and chemokines on day 12 post infection. Our results suggest that SG may protect against weight loss and that SG can regulate intestinal cytokine responses.

## Materials and Methods

### Preparation of *Giardia* Trophozoites

*Giardia intestinalis*, GS (clone H7, ATCC 50581), was used for the experimental infection in mice. The *G. intestinalis* trophozoites were cultured at 37°C in polystyrene screw cap tubes (Nunc) in 10 ml of TYDK media supplemented with 10% heat inactivated fetal bovine serum (Gibco, Thermo Fisher Scientific, MA, USA), bovine bile (12.5 mg/ml), and Ferric ammonium citrate solution (2.2 mg/ml) with the final pH adjusted to 6.8 as described (29). All TYDK medium components were purchased from Sigma-Aldrich (MO, USA) unless stated otherwise. *Giardia* trophozoites were pelleted at 931 × g for 10 min after being kept on ice for 15 min, and re-suspended in ice-cold PBS at 10^6^ parasites per 100 μl, *i.e.* the infectious dose for each mouse.

### Mouse Breeding and Ethical Statement

The serglycin (SG) knockout mouse strain, congenic on the C57BL/6J Taconic genetic background (generation N > 20, which routinely have been backcrossed to original C57BL/6J Taconic mice every second year since 2004), were kept under specific pathogen-free conditions at the Faculty of Veterinary Medicine and Animal Science, SLU, Uppsala, Sweden. Heterozygote (SG^+/−^) mice were mated to produce the groups of young (≈11 weeks old) female and male littermate mice to be used in the experimental infections. All experimental infections were conducted in agreement with the Swedish Animal Welfare Act and granted permission (#C140/15) from the Ethical Committee for Animal Experiments, Uppsala District Court. A maximum of 5 mice per cage were housed in individually ventilated cages (IVCs, ca 501 cm2 Macrolon IIL cages) with aspen shavings for bedding, paper for nesting, and a small house of paper. A 12 h light cycle was used and water and rodent chow were provided *ad libitum*. No antibiotics were included in the diet and the mice were never treated with any drugs.

### Infections and Scoring of Mice, and Sample Collections

Young SG^+/+^, SG^+/−^ and SG^−/−^ littermate female and male mice (in the age range of 7 to 12 weeeks old, with the majority ≈11 weeks old) were infected by oral gavage with 10^6^ *Giardia* trophozoites of the GS isolate in 100 μl PBS in two independent experiments (see **Supplementary Table S1**). As a control, young SG^+/+^, SG^+/−^ and SG^−/−^ littermate mice were oral gavage-challenged with 100 μl PBS only. The clinical scoring was performed in a blinded fashion, *i.e.* the genotypes of infected littermate mice were not known to the assessor until determined at the experimental endpoint. In brief, weight data were recorded before infection (day 0) and then every second to third day post infection (dpi) until infected mice were euthanized at the experimental endpoint, day 12. Feces were collected every second or third day for nested PCR detection of *G. intestinalis*. Blood was sampled at the experimental endpoint and allowed to clot to collect serum. Tail tissue was collected for genotyping of the mice. Small intestines (jejunum and duodenum) were collected at 12 dpi and cut into one-centimeter pieces, and stored at −80^0^C until used. Weight data were pooled from two independent experimental infections, and as we could not find any significant differences in the weight gain between the SG^+/+^ and SG^+/−^ mice in the PBS challenged group or during the challenge infection with *Giardia*, the scored weight changes in the SG^+/+^ and SG^+/−^ mice were also pooled. Since only four SG^−/−^ females were found in the blinded PBS-challenged group we included three uninfected male SG^−/−^ mice in the qPCR analysis.

### Genotyping of SG Mice

Tail tissue samples were heated in 50 μl digestion buffer [0.2M Tris, 0.1M (NH_4_)_2_SO_4_, 0.05M MgCl_2_, 1% beta-mercaptoethanol, 0.5% Triton-X 100, autoclaved Milli Q water] at 95°C for 10 min. To digest the tail tissue and extract mouse genomic DNA Proteinase K (2 μg/ul, #AM2544, Ambion) was added and the samples were incubated overnight at 55°C. The crude extracts were then heated for 10 min at 95°C to inactivate the Proteinase K activity, and centrifuged at 16,200 × g to pellet insoluble material. 0.5 μl of the crude DNA samples was used for the PCR-based genotyping of the SG littermate mice. The following primers were used: Forward 5’-GTC TCT GTT TTC ACA TTC CAC GGC CC-3’, Reverse 5’-GGC ACA AGC AGG GAA CAT TCC GAG C-3’, and Reverse Neo-cassette 5’-GGG CCA GCT CAT TCC TCC CAC TCA TGA TCT-3’, which yielded the expected 315 bp WT and 550 bp KO products. PCR was performed with the KAPA2G Robust HotStart PCR kit (#KK5517, Techtum).

### PCR Assay for Detection of Giardia DNA

Collected fecal samples were treated as described for the tail tissue to recover DNA. *Giardia* DNA was demonstrated using a nested PCR with the following primers targeting beta-giardin: first round: Forward 5’-AAA TNA TGC CTG CTC GTC G-3’ and Reverse 5’-CAA ACC TTN TCC GCA AAC C-3’ and the second round: Forward 5’-CCC TTC ATC GGN GGT AAC TT-3’ and Reverse 5’-GTG GCC ACC CAN CCC GTG CC-3’, yielding a 530 bp product (30). PCR was performed with the KAPA2G Robust HotStart PCR kit (#KK5517, Techtum).

### Sampling and Morphological Assessment of Intestinal Tissues

In brief, 1 cm of the duodenum was collected and fixed in 10% formalin for at least 24 h, embedded in paraffin, and sectioned on a Microtome in 5 μm tissue sections. The tissue sections were mounted on slides and stained with hematoxylin and eosin (H&E). Pathological changes were assessed by light microscopy and the numbers of goblet cells as well as granulocytes were counted in ≥30 villi crypt units (VCU) per mouse intestine.

### Determination of Myeloperoxidase Activity and Neutrophil Elastase Activity in the Intestine

To measure peroxidase activity, *i.e.* myeloperoxidase (MPO) and eosinophil peroxidase (EPO), intestinal tissues were frozen in liquid nitrogen and grinded with a mortar and pestle into a tissue powder. The powdered tissues were resuspended in cold 1% hexadecyl trimethyl ammonium bromide in phosphate buffer (400 μl per 50 mg tissue) with pH 6.0. After snap freezing and thawing three times in liquid nitrogen, the homogenate was centrifuged at 12,000 × g for 15 min at 4°C to obtain the supernatant. Then 10 μl of supernatant was added in 200 μl of substrate solution (50 mmol/L phosphate buffer, 0.4 mg/ml o-phenylenediamine substrate, and 0.05% H_2_O_2_). After 20 min the reaction was stopped by adding 50 μl of 0.4 mol/L H_2_SO_4_, and the absorbance was measured at 490 nm.

To assess neutrophil elastase (NE) activity intestinal tissues were frozen in liquid nitrogen and grinded with a mortar and pestle into a tissue powder. The powdered tissues were re-suspended in 1 ml Hank’s balanced salt solution (HBSS) per 50 mg tissue, and centrifuged at 15,000 × g for 30 min at 4°C. Fifty microliters of the obtained supernatants were incubated with 15 μl of 10 μM elastase substrate aSuc-Ala-Ala-Pro-Val-AMC (#L-1770, Bachem) in 135 μl of the substrate reaction buffer (150 mM NaCl, 100 mM Tris-HCl, 0.1% BSA, 0.05% Tween-20, MilliQ water, pH = 8.5). The optical density (OD) at 405 nm was determined at 0 min and after 60 min and the difference calculated.

### RNA Extraction and qPCR Detection of Chemokine and Cytokine Expressions in the Intestine

Collected intestinal samples from PBS-challenged and *Giardia*-infected mice from experiment #2 were frozen in liquid nitrogen and grinded with a mortar and pestle into a tissue powder. The powdered tissues were resuspended in TRIzol^®^ (Thermofisher) and RNA extraction performed according to the manufacturer’s instructions. DNase I treatment of extracted RNA was incorporated into the procedure to remove traces of genomic DNA. The quality of extracted total RNA was assessed by measuring the 260/280 and 260/230 ratios using a NanoDrop 1000 Spectrophotometer (Thermo Fisher Scientific). The relative intestinal expression of the following chemokines, cytokines, and synthases were analyzed by qPCR: CCL2, CCL20, CXCL1, CXCL2, CXCL3 and IL-1, IL-2, IL-4, IL-5, IL-6, IL-9, IL-10, IL-12, IL-17a, IL-17c, IL-25, IL-33, IFN-γ, TGF-β, TNF-α, as well as NOS1 and NOS2. All qPCR primer pairs were designed in-house using the Primer-BLAST software at the National Center for Biotechnology Information (see **Supplementary Table S2** for the primer pairs). One μg of total RNA was reverse transcribed to cDNA using the RevertAid H Minus First Strand cDNA Synthesis Kit (Thermo Scientific) according to the manufacturer’s instructions. Maxima SYBR Green/ROX qPCR Master Mix (Thermo Scientific) was used for the qPCR and the expression of GAPDH was used for normalization according to guidelines of AB Applied Biosystems (Step One Plus Real Time PCR systems). All qPCR reactions were run in a Step One Plus Real Time PCR machine (Applied Biosystems, Thermo Fisher Scientific, MA, USA) using the following cycling conditions: activation of polymerase at 95°C (15 s), annealing at 60°C (30 s), and extension at 72°C (30 s) followed by melt curve analysis as part of the default run settings. The fold change in gene expression between the PBS-challenged and the *Giardia*-infected SG^+/+^ and SG^−/−^ mice was then calculated using the Livak-method (2^−ΔΔCT^) (31). Since, after genotyping, only four SG^−/−^ females were found in the PBS-challenged group three uninfected male SG^−/−^ mice were included in the PBS-challenged SG^−/−^ group for the qPCR analysis.

### ELISA Assay for IL-6 Detection

The concentration of IL-6 was determined in serum samples from mice in experimental infections #1 and #2, using a mouse IL-6 ELISA developmental kit (#900-T50, PeproTech) according to the supplier´s protocol.

### Statistical Analysis

GraphPad Prism 9 was used for the statistical analysis of the collected data. To compare differences between groups of infected SG^+/+^ and SG^−/−^ littermate mice and PBS controls, weight curves were analysed using the parametric two-way ANOVA Mixed-effect model with uncorrected Fisher’s LSD. The accumulated weight means, intestinal cell counts, and serum IL-6 levels were analysed with parametric one-way ANOVA and uncorrected Fisher’s LSD. For the fold change calculated on the qPCR data the non-parametric Kruskal-Wallis with uncorrected Dunn’s test was used. P values of <0.05 were considered as significant.

## Results

### Serglycin-Deficient Mice Show Reduced Weight Gain During Infection With *Giardia intestinalis*

**Figure 1:**
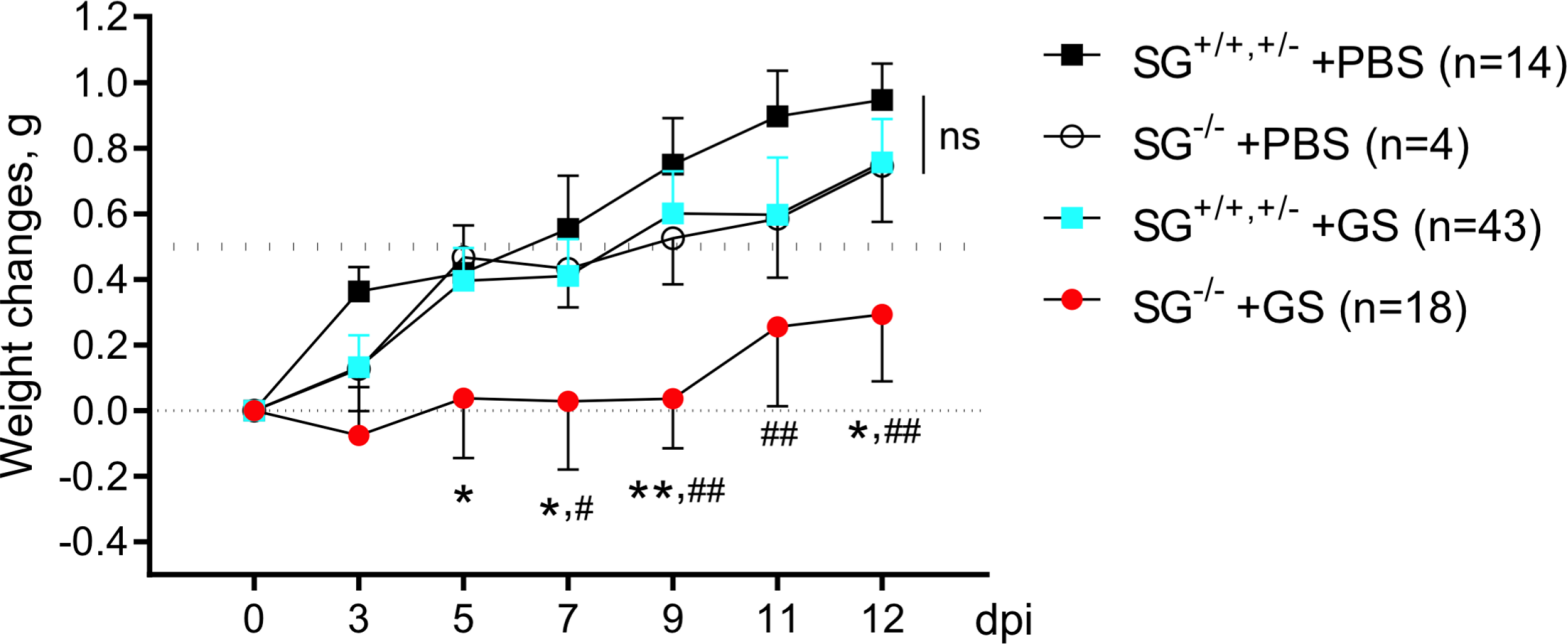
Weight scoring in and SG^+/+, +/-^ and SG^-/-^ mice infected with *Giardia intestinalis*. Young (≈11 weeks old) congenic SG^+/+^, SG^+/-^, SG^-/-^ C57Bl/6 littermate female and male mice without antibiotic treatment were challenged by oral gavage with PBS or with 10^6^ *Giardia intestinalis* trophozoites in PBS Weight data and feces were collected every second or third day until endpoint, and weight changes were normalized to the Initial weight at day 0. Weight data of the PBS-challenged SG^+/+,+/-^(n = 14) and SG^-/-^ (n = 4) mice and pooled weight data of all *Giardia intestinalis* infected SG^+/+,+/-^ (n = 43) and SG^-/-^ (n = 18) mice from two independent infection experiments are shown (see **Supplementary S1**). For comparisons between all groups and days a Mixed-effect analysis was performed. ns, non-significant, P values * <0.05 and **<0.01 for SG^-/-^ +GS *vs* SG^+/+,+/-^ +GS mice, and ^#^ <0.05 and ^##^ <0.01 for SG^-/-^ +GS *vs* SG^+/+,+/-^ +PBS mice are indicated.

To study the putative role of the proteoglycoan SG during *Giardia* infection we oral gavage infected wild-type and SG-deficient littermate mice with the human *G. intestinalis* assemblage B isolate GS (10^6^ trophozoites per mice). Two independent experimental infections, #1 and #2 (**Figures S1A, B**), showed similar results why the weight data were pooled. As a control, oral gavage PBS-challenge of SG-competent and SG-deficient littermate mice was performed once. All four groups of mice significantly gained weight after challenge over the 12 day experimental period (**Figure 1**) , although the accumulated mean weight of the infected SG^−/−^ mice was significantly lower as compared with the other groups (**Figure S1C**). The ≈11 week old SG-competent mice showed a 0.5 g weight gain between 7 and 9 days efter challenge, and the challenge with *G. intestinalis* caused no major reduction in weight gain in the SG^+/+,+/−^ mice, as compared with the PBS-challenged SG^−/−^ and SG^+/+,+/−^ mice (**Figure 1**). In contrast, infected SG^−/−^ mice showed a significantly reduced weight gain as compared to infected SG^+/+,+/−^ mice (at 5, 7, 9, and 12 dpi) and PBS-challenged SG^+/+,+/−^ mice (at 7, 9, 11, and 12 dpi) (**Figure 1**). In experimental infection #1 infected SG^−/−^ mice had a significantly reduced weight gain (at 5, 7, 9, and 12 dpi) as compared to the PBS-challenged SG^−/−^ mice, an effect that was lost in experimental infection #2 (**Figures S1A, B**). In feces collected from day 3 to 12 dpi, a nested PCR identified *G. intestinalis* DNA in all infected mice from 8 dpi and onward and no major differences in the level of *G. intestinalis* DNA in the fecal material was seen between infected SG^−/−^ and SG^+/+,+/−^ mice (**Figure S2** and data not shown). Thus, SG-deficiency has a negative effect on weight gain during *G. intestinalis* infections but it cannot be correlated to the amount of parasite DNA in fecal material.

### Increased Goblet Cell and Granulocyte Counts but Decreased Neutrophil Elastase Activity, With No Major Histopathological Changes of the Small Intestine, in *Giardia*-Infected SG^+/+^ and SG^−/−^ Mice

**Figure 2:**
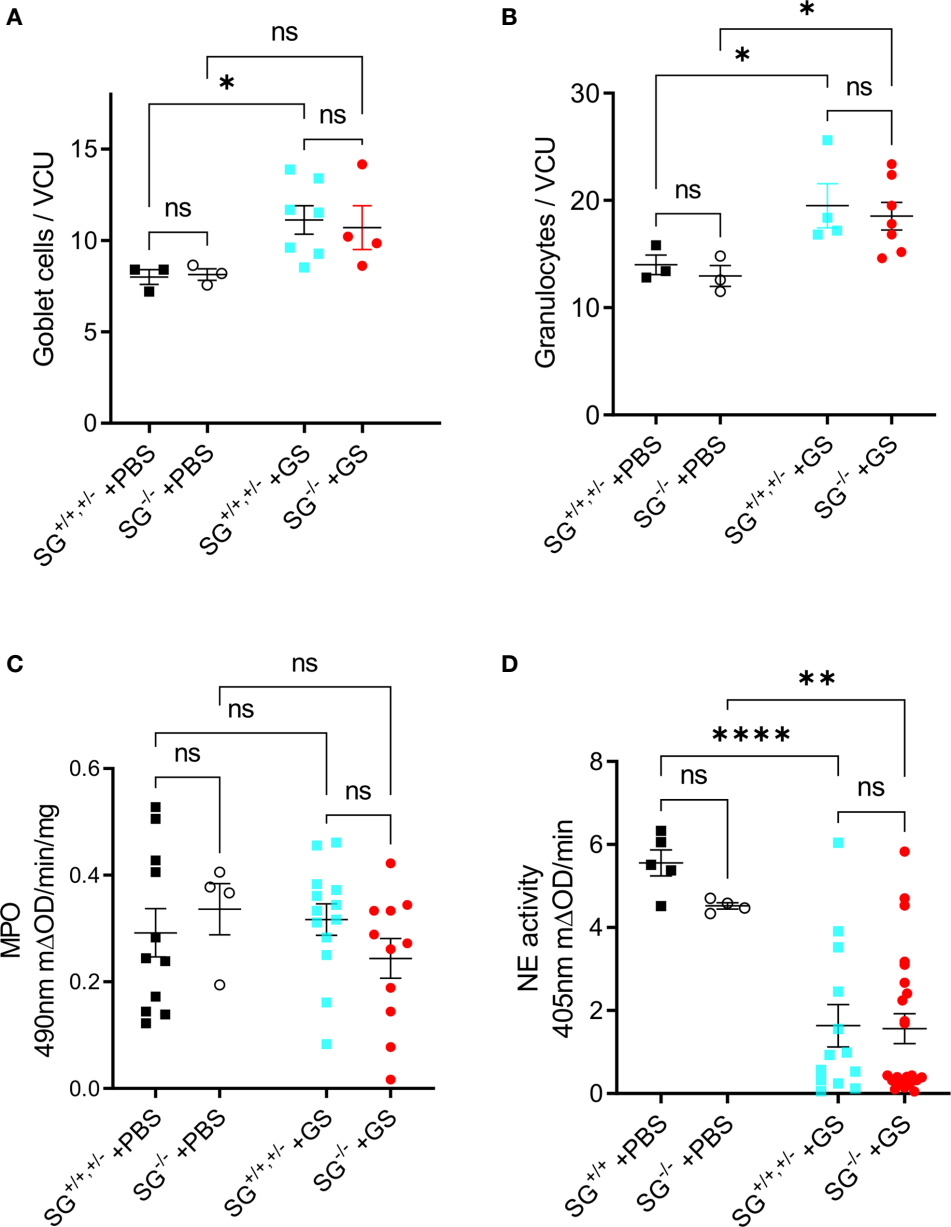
Infection with *Giardia intestinalis* increases goblet cell and granulocyte counts in the small intestine but inhibits neutrophil elastase (NE) activity. **(A)** Goblet cells and **(B)** granulocytes were counted per villi crypt unit (VCU) on H&E-stained sections from PBS-challenged and Giardia-infected mice. **(C)** Intestinal MPO activity (substrate o-phenylenedamine) and **(D)** neutrophil elastase. (NE) activity (substrateL-1770) in PBS-challenged and Giardia-infected SG^+/+^ and SG^-/-^ littermate mice. ns, non-significant, P values * <0.05, ** <0.01, *** <0.0001.

To start to delineate why the *Giardia*-infected SG^−/−^ mice showed a delayed and reduced weight gain we first studied intestinal morphology and granulocyte infiltration in infected mice. The experimental infection with *G. intestinalis* caused no major histopathological changes in young infected SG^+/+^ and SG^−/−^ littermate mice (experiment #2, n^+/+^ = 4, n^−/−^ = 7) as compared to the PBS-challenged SG^+/+^ and SG^−/−^ littermate mice (n^+/+^ = 3, n^−/−^ = 3) (**Figures S3A, B**). However, at 12 dpi the infection caused significantly increased goblet cell counts (**Figure 2A**) and granulocyte cell counts (**Figure 2B**), independently of SG. The H&E staining of intestinal tissues may poorly resolve the ratio of neutrophilic and eosinophilic granulocytes, therefore we next assessed the intestinal activities of eosinophil peroxidase (EPO) and myeloperoxidase (MPO) expressed by neutrophils and monocyte/macrophages, as well as the major neutrophil-derived enzyme neutrophil elastase (NE). Whereas both peroxidase and NE activity levels were found to be SG-independent in challenged mice, the peroxidase activity levels were not increased (**Figure 2C**), and the NE activity significantly decreased (**Figure 2D**), as compared with PBS-challenged mice. Together, this suggests that SG has no major role in granulocyte recruitment and the activity of MPO and NE during the *Giardia*-infection.

### Significantly Lower Serum IL-6 Levels and Intestinal Transcriptional IL-6 Levels in *Giardia*-Infected Serglycin-Deficient Mice

**Figure 3:**
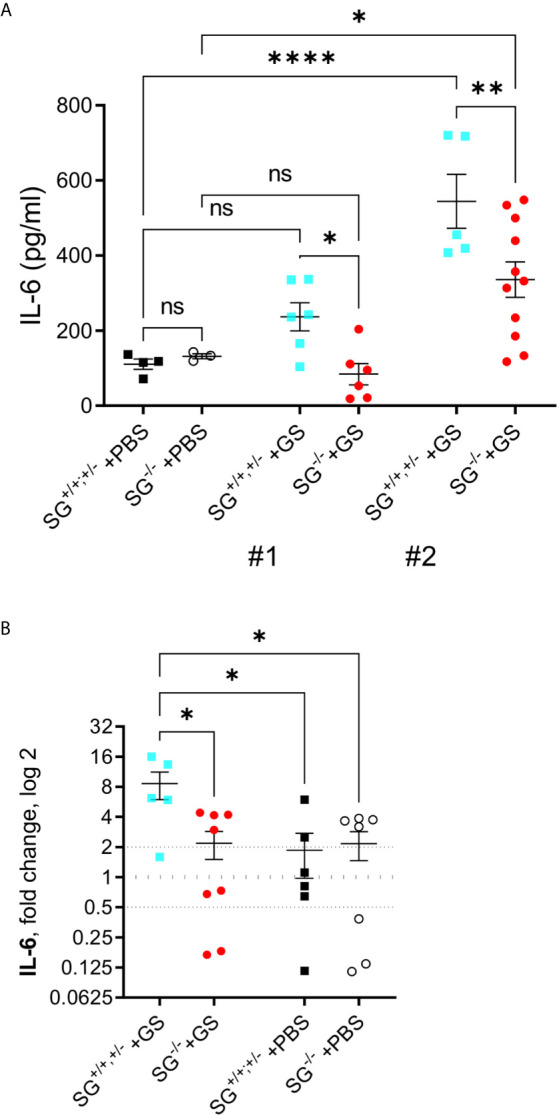
Serum levels of IL-6 and intestinal expression levels of the proinflammatory cytokine IL-6 in SG^+/+^ and SG^-/-^ littermate mice infected with *Giardia intestinalis*. **(A)** Serum IL-6 levels in from experimental infections #1 and #2. Relative intestinal expression levels determined by qPCR of **(B)** IL-6. Fold changes were calculated after normalization to GAPDH_ Dotted lines at 0.5, 1, and 2 indicates down-regulation, no fold change, and up-regulation, respectively. ns, non-significant, P values * <0.05 ** <0.01, and **** <0.0001 between groups of mice are indicated.

The pro-inflammatory cytokine IL-6 has been shown to play a significant role in the host control of *Giardia* infections (32) and in addition SG-dependent serine-proteases have been shown to be involved in the degradation of IL-6 (33). With this in mind the serum IL-6 levels was determined with ELISA in the two independent experimental infections [experiment #1 (SG^+/+^ n = 6, SG^−/−^ n = 6) and #2 (SG^+/+^ n = 5, SG^−/−^ n = 11)]. While experiment #1 showed a non-sigificant increase in IL-6 levels in comparison to PBS-challenged mice (SG^+/+^ n = 4, SG^−/−^ n = 3) the IL-6 levels were significantly increased in experiment #2, although the infected SG^−/−^ mice showed significantly lower levels of serum IL-6 as compared to the infected SG^+/+^ mice in both experiments (**Figure 3A**). To further explore this difference we next evaluated the expression levels of IL-6 with qPCR, in intestinal tissues collected from experiment #2. Compared with the non-infected/PBS-challenged mice the *Giardia*-infection caused a significantly increased expression of IL-6 in the SG-competent mice, while infected SG^−/−^ mice showed significantly reduced intestinal expression levels of IL-6 compared with infected SG^+/+,+/−^ mice (**Figure 3B**).

### Expression Levels of Interleukins and Cytokines As Well As Nitric Oxide Synthases in the Small Intestine of *Giardia*-Infected SG^+/+^ and SG^−/−^ Mice

**Figure 4:**
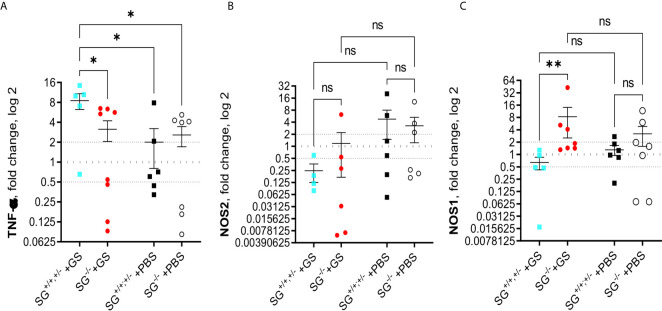
Intestinal expression levels of TNF-α and nitric oxide synthases (NOS) in SG^+/+^ and SG^-/-^ littermate mice infected with *Giardia intestinalis*. Relative intestinal expression levels of **(A)** TNF-α, **(B)** inducible NOS2, and **(C)** ubiquitously expressed NOSI, determined by qPCR. Fold changes were calculated after normalization to GAPDH. Dotted lines at 0.5, 1, and 2 indicates down-regulation, no fold change, and up-regulation, respectively. ns= non-significant, P values * <0.05, ** <0.01.

To further explore potential effects causing the reduced weight gain in *Giardia*-infected SG^−/−^ mice we continued analyzing the transcriptional levels of immune related genes in the small intestines of infected mice, collected in experiment #2. First we studied cytokines that earlier have been shown to be up-regulated during *Giardia* infections in mice, *i.e.* IL-1, IL-2, IL-4, IL-5, IL-9, IL-10, IL-12, IL-17a, IL-17c, IFN-γ, TGF-β, and TNF-α. At 12 dpi the infected SG^+/+,+/−^ mice showed significantly increased expression of TNF-α as compared with the other mice groups (**Figure 4A**), whereas only minor changes in the expression of the other cytokines as compared with the PBS-challenged mice was noted (**Figures S4A–K**). However, a non-significant trend for increased expression of several of the evaluated cytokines was noted in the infected mice, and the accumulated ranking means of fold changes of all the “A to K” cytokines were significantly increased in the infected mice as compared with PBS-challenged mice (**Figure S4L**). Furthermore, the inducible nitric oxid synthase 2 (NOS2), which is expressed by several immune cells, remained indifferent with a non-significant down-regulation in the infected mice (**Figure 4B**). In contrast, the level of NOS1 was significantly upregulated in the infected SG^−/−^ mice (**Figure 4C**), suggesting that SG may be involved in the regulation of the respiratory burst produced by resident intestinal cells.

### Expression Levels of Alarmins and Chemokines in the Small Intestine of *Giardia*-Infected SG^+/+,+−^ and SG^−/−^ Mice

**Figure 5:**
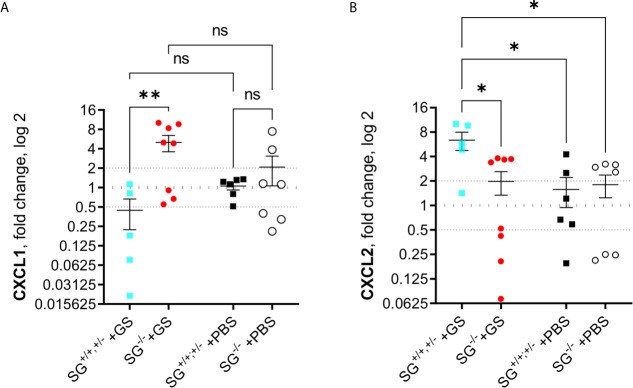
Intestinal expression levels of chemokines in *Giardia intestinalis*-infected SG^+/+^ and SG^-/-^ littermate mice. Relative expression levels at day 12 post infection of **(A)** CXCL1 and **(B)** CXCL2 determined by qPCR. Fold changes were calculated after normalization to GAPDH. Dotted lines at 0.5, 1, and 2 Indicates down-regulation, no fold change, and up-regulation, respectively. ns, non-significant, P values * <0.05, ** <0.01 are Indicated.

Since the alarmins IL-25 and IL-33 have been shown to be important during intestinal worm infections and the chemokines CCL2, CCL20, CXCL1, CXCL2, and CXCL3 were found to be up-regulated in human epithelial cells during *Giardia* infection, we finally assessed the expression levels of these factors in the small intestines of infected mice at 12 dpi. While there was no major changes of IL-25, IL-33, CCL2, CCL20, and CXCL3 (**Figures S5A–E**), or in accumulated ranking means of the “A to E” factors (**Figure S5F**), there was a significant SG-dependent regulation of CXCL1 (**Figure 5A**) and CXCL2 (**Figure 5B**), with more CXCL1 and less CXCL2 expressed in the infected SG^−/−^ mice. Thus, there is an effect of SG on the intestinal expression of a limited set of chemokines during *Giardia* infection.

## Discussion

We here addressed if and how the targeted deletion of the proteoglycan SG, which in the small intestine is expressed by many residing cells as well as in recruited immune cells, affects weight gain and intestinal immune responses during an infection with the non-invasive protozoan parasite *G. intestinalis*. Young (≈11 weeks old) SG^+/+^, SG^+/−^, SG^−/−^ littermate mice were infected by gavage and scored in a series of completely blinded experiments. It is now well-documented that infection with *G. intestinalis* influences the growth and weight gain of young humans and animals, including experimentally infected mice and gerbils (7, 8, 34–37). However, most of the earlier experimental *Giardia* infections with the *Giardia* GS H7 isolate in young wild-type mice around 10 weeks of age have shown spontaneous clearance after 2–3 weeks and the weight gain is often not affected (8, 38). Interestingly, we found that SG protects against reduced weight gain induced by the *G. intestinalis* infection, whereas young mice deficient in the mast cell chymase mMCP-4 gained weight similar to wild-type mice (38). This suggests a role for SG-dependent immune effectors, other than mMCP-4, in the *Giardia*-induced growth defects. The SG-dependent effect on growth in *Giardia*-infected mice can be immune related but also related to changes in the metabolism in lipids and other nutrients, changes in intestinal absorption, appetite-leptin expression, and microbiota composition and this is discussed further below.

To further elucidate the contribution of SG proteoglycans in weight gain during a *G. intestinalis* infection we evaluated tissue morphology and molecular processes in the small intestine. Although the intestinal morphology of *Giardia*-infected SG^−/−^ and SG^+/+^ mice displayed no major histopathological changes as compared with PBS-challenged mice, the infection caused similarly increased goblet cell and granulocyte counts in the villus crypt unit (VCU) of the SG^−/−^ and SG^+/+^ mice. The granulocyte counts at 12 dpi in the young mice was similar to what we previously found in mature adult mice at 13 dpi (38), suggesting that granulocyte recruitment during a *G. intestinalis* infection may occur independent of the age of the mice. To determine if the observed increase in granulocytes were of neutrophilic or eosinophilic origin we measured peroxidase activity as well as the activity of the neutrophil derived enzyme NE. In neutrophilic granulocytes MPO produces oxygen radicals that could destroy engulfed bacteria and MPO activity is frequently used as a marker for recruitment of neutrophils to the infected tissue (39). However, the substrate used in the current study will also detect eosinophilic peroxidase (EPO) activity. Although the experimental *Giardia*-infection could be considered as “mild” in the young mice, as reflected by the low increase of granulocytes, activated eosinophils and neutrophils will export their granule content and thus, some activity are expected to be lost out in the lumen. Interstingly, while the MPO/EPO activity was similar between PBS and *Giardia*-challenged mice suggesting luminal export, the *G. intestinalis* infected mice showed a significantly reduced activity of NE, independently of SG. This finding is in line with recent *in vitro* data suggesting that *G. intestinalis* may produce and secrete inhibitors to eliminate the potentially harmful effects of NE (38). NE is protectively involved in a wide range of different infection, and will degrade extracellular matrix components and cell surface molecules during tissue injury and inflammation (40). Interestingly, in neutrophils *in vitro* differentiated from bone marrow cells of the SG knockout mice a complete lack of NE was reported (26). However, we recently showed that the intestinal levels of NE activity increased significantly in SG-deficient mice infected with *Thichinella spiralis* (28) and, in the present study we found detectable NE activity in the small intestines of the PBS-challenged SG^+/+^ and SG^−/−^ mice and that the activity was only slightly lower in the SG^−/−^ mice than in the SG^+/+^ mice. This suggests that SG may be important for the production and granular storage of NE during neutrophil differentiation *in vitro*, but not for the production and secretion of NE from intestinal neutrophilic granulocytes *in vivo*. Thus, the slightly increased expression of IL-5 in combination with indifferent level of MPO activity and the reduced NE activity suggests that mainly eosinophilic granulocytes may account for the observed increase of granulocyte numbers in the small intestines of the infected SG^−/−^ and SG^+/+^ mice, a suggestion that warrants further investigation.

To alert the intestinal immune responses towards the *G. intestinalis* infection secretion of alarmins such as IL-25 and IL-33, and chemokines such as CCL2, CCL20, CXCL1, CXCL2, and CXCL3, likely initiate the early recruitment of innate immune cells. Later a mixed Th1/Th2/Th17 immune reaction should follow with increased expression of several cytokines such as TNF-α, IFN-γ, IL-1, IL-2, IL-4, IL-5, IL-6, IL-8, IL-9, IL-10, IL-12, IL-17, and IL-23 (12, 19, 32, 41–47). In addition, the activated intestinal tissue and recruited immune cells starts production of nitric oxide and other oxygen species that can destroy invading microorganisms. In the current model, at day 12 of the *G. intestinalis* infection significantly changed expression of IL-6, TNF-a, CXCL1, CXCL2, and NOS1 was noted in the small intestines of young mice. In addition, IL-1, IL-5, IL-12, IFN-γ, IL-25, and CCL2 showed a tendency for changed expression levels in infected mice. The chemokines CXCL1 and CCL2 have earlier been shown to be highly up-regulated by human intestinal epithelial cells (11) whereas earlier studies with mouse intestinal cell lines or experimental infections failed to detect up-regulation (48). CXCL1, which is a potent mediator of neutrophil recruitment will require proteoglycan GAGs, *e.g.* heparan sulphate, for their functionality during interaction with the receptor CXCR2 on incoming granulocytic cells (49). Interestingly, the deletion of SG significantly increased the expression level of the chemokine CXCL1 in the small intestine, suggesting that lack of SG may cause a compensatory upregulation of CXCL1. However, as we did not find a SG-dependent difference in the granulocyte counts or NE activity other proteoglycans in the small intestine likely provide sufficient amounts of GAGs for the CXCL1-dependent recruitment of granulocytes.

Both IL- 6 and TNF-α contributes to the expulsion of *Giardia* (13, 32, 41, 43, 44, 50) and Li et al. reported that neuronal nitric oxide synthase (NOS) 1 was essential for the elimination of *G. intestinalis* in young (5 to 8 weeks old) female mice at 12 dpi, whereas the expulsion was found to be independent of IL-6-induced expression of NOS2 (51). Interestingly, we found a significantly decreased IL-6 expression level and increased intestinal expression level of NOS1 in the infected SG^−/−^ mice, suggesting a compensatory mechanism of NOS1 *versus* IL-6 in the SG-deficient mice. Thus, the increased activation of NOS1 could partly explain the reduced weight gain observed in the SG^−/−^ mice, as the nitric oxide formed would lead to an aggressive intestinal milieu. However, the role of SG for intestinal expression of IL-6 and NOS1 requires further studies.

The observed effects of a reduced weight gain in *G. intestinalis* infected SG deficient mice is most likely the result of several interacting factors, not only immune-related. An earlier mouse model of *Giardia* induced growth delay used 5-week-old mice and cysts from the H3 *G. intestinalis* isolate, but decreased growth was not seen until 35 days (8). Malnutrition by a low-protein diet in these animals increased the growth defects and the effects were seen already after 13 days, similar to what we see in our SG model, and it reduced the expression of the cytokines IL-4 and IL-5 compared to *Giardia* infected well-nourished mice (8). Thus, there are connections between protein metabolism and immune responses during *Giardia* infections and this can be studied further in the SG-model. Another, more recent study using a *G. intestinalis* GS infection model in neonatal mice resulted in persistent infections (20 weeks) and a reduced growth and weight gain (52). *Giardia* preferentially infects the upper small intestine where bile is plentiful and it is part of the growth medium used to grow *Giardia* trophozoites *in vitro* (53). The effects with reduced weight gain in the neonatal mice infected with *G. intestinalis* were associated with changes in the bile acid and lipid metabolism and changes in the composition of the microbiota (52). Serglycin-deficient mice have an impaired lipid metabolism (54) and a changed bile and lipid metabolism in the intestine, potentially with associated changes in the intestinal microbiota, can be an important factor in the reduced weight gain during *Giardia* infections in SG-deficient mice. It is now clear that the intestinal microbiota is very important during *Giardia* infections and antibiotic treatments have sever effects on the infection dynamics (55). The intestinal microbiota affect IgA production, antimicrobial peptides, intestinal motility, and bile and lipid metabolism during *Giardia* infections (55). In our SG-model we use non-antibiotic treated 10-week old mice and the negative aspects with this is lower levels of infection and reduced immune responses but it also means that we can study the effects of lipid metabolism and microbiota on weigth gain during giardiasis. Further studies in the SG-deficient mouse model can reveal how these factors interact during *Giardia* infections.

In summary, the *G. intestinalis* infection in young littermate SG^+/+^ and SG^−/−^ mice caused a reduced weight gain in the SG^−/−^ mice and SG-dependent changes in the intestinal mRNA levels of NOS1, CXCL1, CXCL-2, TNF-α, and IL-6, as well as a significant SG-dependent change in the IL-6 serum protein levels. Thus, our results suggests a role for SG in both innate and adaptive intestinal immune responses towards the *G. intestinalis* infection. The SG^−/−^ mouse strain provides not only an interesting model for elucidating the roles of the proteoglycan SG and SG-dependent inflammatory mediators in the intestinal host responses towards intestinal parasites like *G. intestinalis* but also as a unique model for studies of weight gain during infections by intestinal protozoa.

## Data Availability Statement

The original contributions presented in the study are included in the article/**Supplementary Material**. Further inquiries can be directed to the corresponding author.

## Ethics Statement

The animal study was reviewed and approved by the Ethical Committee for Animal Experiments, Uppsala District Court granted the permission (#C140/15), and all experimental infections were conducted in agreement with the Swedish Animal Welfare Act.

## Author Contributions

Conceptualization, SS and MÅ. Formal analysis, ZL, DP, and MÅ. Funding acquisition, SS and MÅ. Investigation, ZL, DP, and MÅ. Project administration, MÅ. Resources, SS and MÅ. Supervision, SS and MÅ. Visualization, ZL and MÅ. Writing—original draft, ZL, DP, SS, and MÅ. Writing—review and editing, ZL, DP, SS, and MÅ. All authors contributed to the article and approved the submitted version.

## Funding

This study was in part funded by Vetenskapsrådet VR-M, 2012-03364 (awarded to SS) and 2011-03533 (awarded to MÅ).

## Conflict of Interest

The authors declare that the research was conducted in the absence of any commercial or financial relationships that could be construed as a potential conflict of interest.
