# Supplementary figures and images for: Serglycin-Deficiency Causes Reduced Weight Gain and Changed Intestinal Cytokine Responses in Mice Infected With Giardia intestinalis
Source: Front Immunol. 2021 Jul 8;12:677722. doi: 10.3389/fimmu.2021.677722 (PMC8316049; doi:10.3389/fimmu.2021.677722)

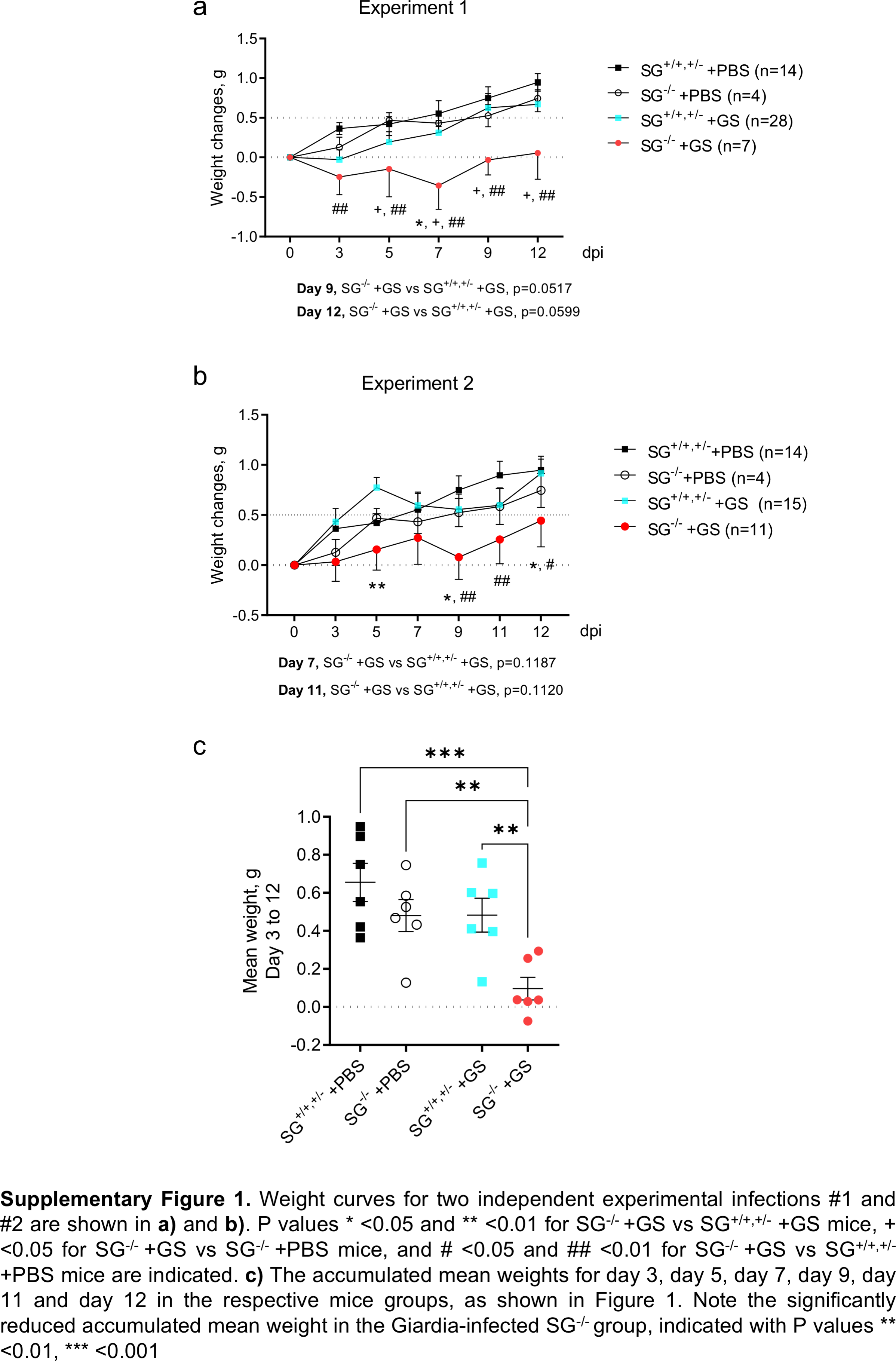

Supplement: Supplementary file 1 [file Image_1.tif]

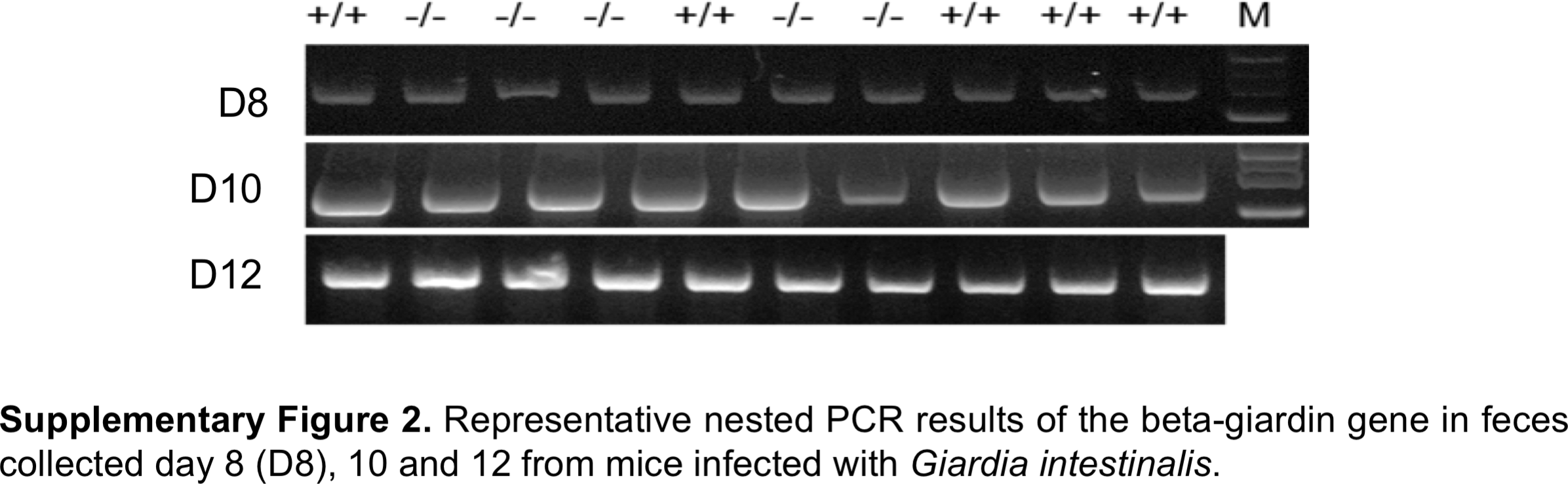

Supplement: Supplementary file 2 [file Image_2.tif]

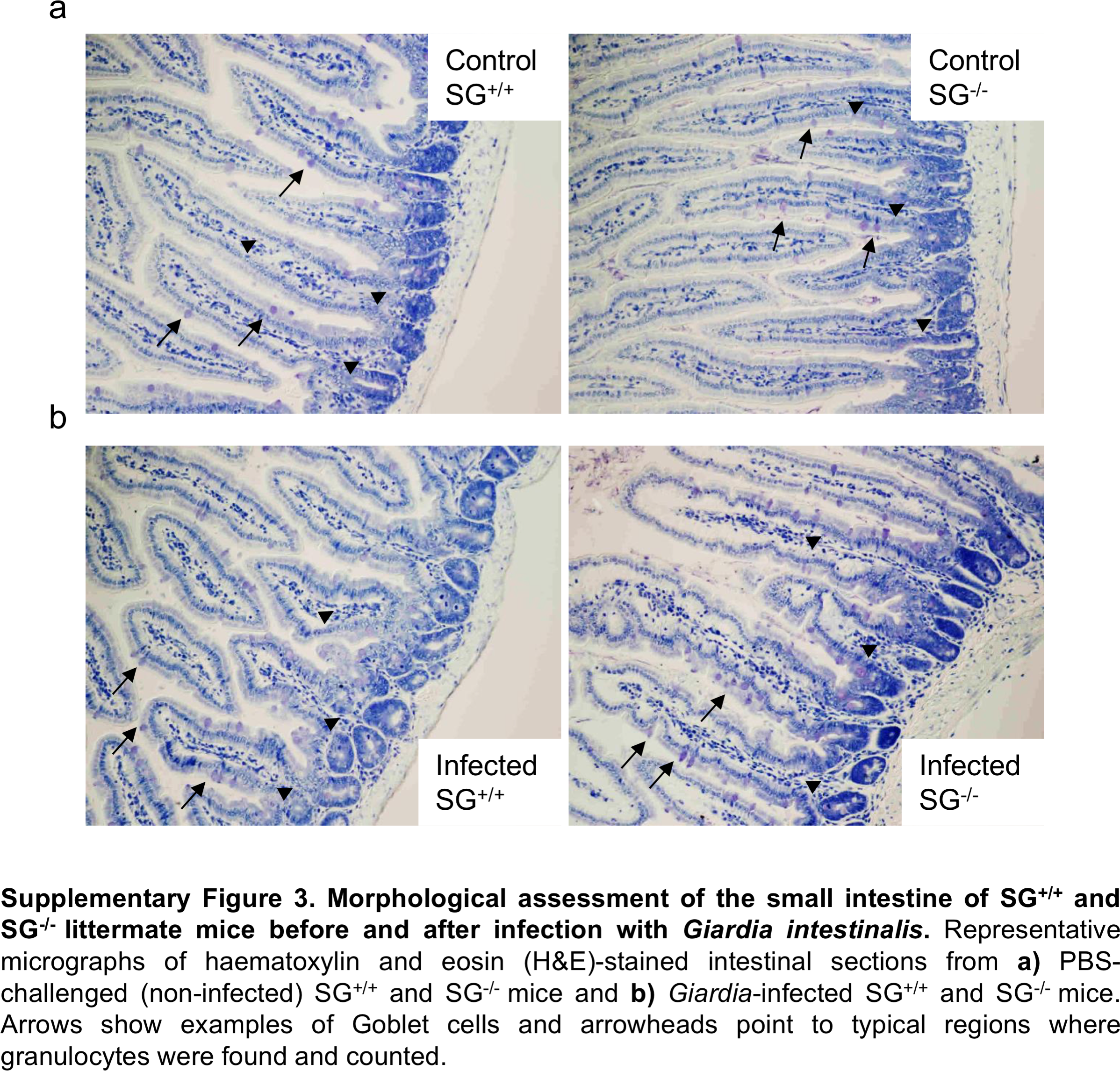

Supplement: Supplementary file 3 [file Image_3.tif]

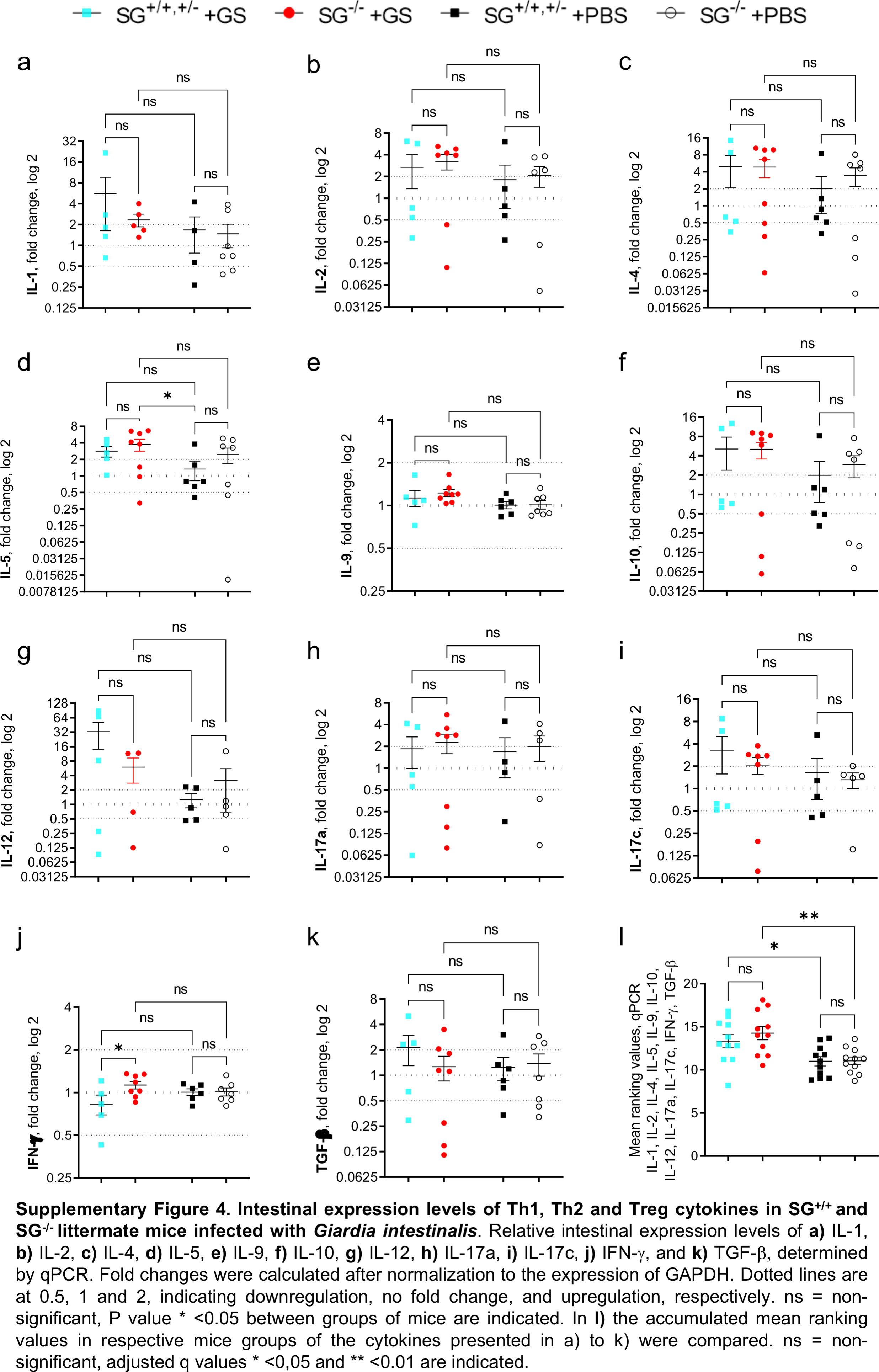

Supplement: Supplementary file 4 [file Image_4.tif]

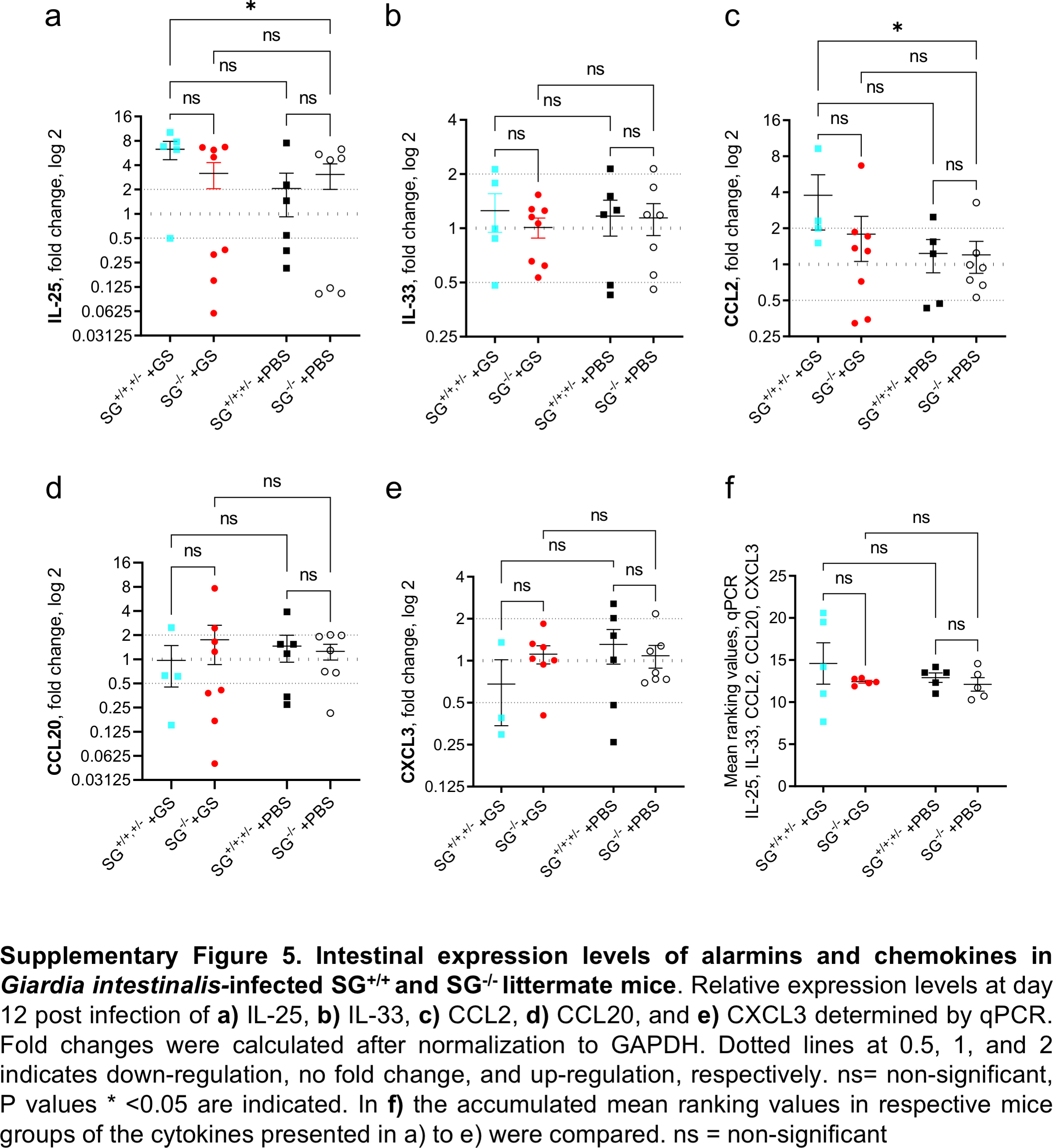

Supplement: Supplementary file 5 [file Image_5.tif]
