# Supplementary material for: Serglycin-Deficiency Causes Reduced Weight Gain and Changed Intestinal Cytokine Responses in Mice Infected With Giardia intestinalis
Source: Front Immunol. 2021 Jul 8;12:677722. doi: 10.3389/fimmu.2021.677722 (PMC8316049; doi:10.3389/fimmu.2021.677722)
Supplement: Supplementary file 6 [file Table_1.docx]

**Supplementary Table 1. Experimental infections in congenic SG-competent and SG-deficient C57Bl/6 littermate mice with the *Giardia* GS-isolate.** Age matched littermate female (F) and male (M) mice without antibiotic treatment were infected by oral gavage with 10^6^ *Giardia intestinalis* trophozoites. The control group were gavage-challenged with PBS.

| **Experiments** | **Sex** | **Number of mice** | **Age (weeks)** | **Endpoint days** |
| --- | --- | --- | --- | --- |
| # 1 | F | +/+ n=2, +/- n=6, -/- n=2 | 7-12 | 12 |
|  | M | +/+ n=3, +/- n=14, -/- n=5 |  |  |
|  |  |  |  |  |
| # 2 | F | +/+ n=4, +/- n=6, -/- n=6 | 11 | 12 |
|  | M | +/+ n=2, +/- n=3, -/- n=5 |  |  |
|  |  |  |  |  |
| control | F | +/+ n=2, +/- n=4, -/- n=4 | 11 | 12 |
|  | M | +/+ n=3, +/- n=5, -/- n=0 |  |  |
