# Supplementary material for: Serglycin-Deficiency Causes Reduced Weight Gain and Changed Intestinal Cytokine Responses in Mice Infected With Giardia intestinalis
Source: Front Immunol. 2021 Jul 8;12:677722. doi: 10.3389/fimmu.2021.677722 (PMC8316049; doi:10.3389/fimmu.2021.677722)
Supplement: Supplementary file 7 [file Table_2.docx]

**Supplementary Table 2.** Primer pairs used for the qPCR evaluation.

| **GENE** | **DIRECTION** | **SEQUENCE** |
| --- | --- | --- |
| GAPDH | forward | CAAGCTCATTTCCTGGTATGACAAT |
|  | reverse | CTCTCTTGCTCAGTGTCCTTGC |
| IL-6 | forward | TGGGACTGATGCTGGTGAC |
|  | reverse | CACAACTCTTTTCTCATTTCCACG |
| TNF-α | forward | ACGGCATGGATCTCAAAG |
|  | reverse | TGGGAGTAGACAAGGTACAACC |
| IFNγ | forward | CACACCTGATTACTACCTTCTTCAG |
|  | reverse | GACTCCTTTTCCGCTTCCT |
| NOS1 | forward | AGAATGGGGAGAAATTCGGC |
|  | reverse | GACGCTGTTGAATCGGACCT |
| NOS2 | forward | AACAATACAAGATGACCCTAAGAG |
|  | reverse | TCTGGAACATTCTGTGCTG |
| IL-25 | forward | GCTCCAGTCAGCCTCTCTC |
|  | reverse | CTGCTCACCAGTCACAGGT |
| IL-33 | forward | TCTGCCCCTTCTTTGGTT |
|  | reverse | GGGAGTAGGAGAGCCGTTAC |
| CXCL-1 | forward | ACCGAAGTCATAGCCACACTCA |
|  | reverse | CTCCGTTACTTGGGGACACCT |
| CXCL-2 | forward | ATACTGAACAAAGGCAAGGCTAACTG |
|  | reverse | CTCAGACAGCGAGGCACATC |
| CXCL-3 | forward | CAGACAGAAGTCATAGCCACTCTCA |
|  | reverse | AGCAGGTAAAGACACATCCAGACAC |
| CCL-2 | forward | CACTCACCTGCTGCTACTCATTC |
|  | reverse | GGTGCTGAAGACCTTAGGGC |
| CCL-20 | forward | CCTCTCGTACATACAGACGCCTC |
|  | reverse | ACACACTATGATGCAATATGAATCAACTT |
| IL-2 | forward | TGTAAAACTAAAGGGCTCTGACA |
|  | reverse | AGAAAGTCCACCACAGTTGCT |
| IL-4 | forward | GCAACGAAGAACACCACAGAG |
|  | reverse | GAAGCACCTTGGAAGCCCTA |
| IL-5 | forward | GAAATACATTGACCGCCAAAAAGT |
|  | reverse | GCCTCAGCCTTCCATTGC |
| IL-9 | forward | CCTTGCCTCTGTTTTGCTCT |
|  | reverse | ATCATCAGTTGGGACGGAGAG |
| TGF-b | forward | CTGCTGACCCCCACTGATAC |
|  | reverse | AAAGCCCTGTATTCCGTCTCC |
| IL-10 | forward | CCTGGTAGAAGTGATGCCCC |
|  | reverse | ATTCAAATGCTCCTTGATTTCTGG |
| IL-1  (both variants) | forward | CTGACTTGTTTGAAGACCTAAAGAACTGT |
|  | reverse | GAGATGGTCAATGGCAGAACTG |
| IL-12  (both variants) | forward | GTCAATCACGCTACCTCCTCT |
|  | reverse | CGGGACTGGCTAAGACAC |
| IL-17a | forward | TCAGACTACCTCAACCGTTCC |
|  | reverse | CTATCAGGGTCTTCATTGCG |
| IL-17c | forward | GAGATATCGCATCGACACAGA |
|  | reverse | CATCCACGACACAAGCATT |
